# Genotype‐specific effects of ericoid mycorrhizae on floral traits and reproduction in *Vaccinium corymbosum*

**DOI:** 10.1002/ajb2.1372

**Published:** 2019-11-01

**Authors:** Alison K. Brody, Benjamin Waterman, Taylor H. Ricketts, Allyson L. Degrassi, Jonathan B. González, Jeanne M. Harris, Leif L. Richardson

**Affiliations:** ^1^ Department of Biology University of Vermont Burlington VT 05405 USA; ^2^ University of Vermont Extension Center for Sustainable Agriculture Burlington VT 05401 USA; ^3^ Waterman Orchards Johnson VT 05656 USA; ^4^ Gund Institute for Environment University of Vermont Burlington VT 05405 USA; ^5^ Rubenstein School of Environment and Natural Resources University of Vermont Burlington VT 05405 USA; ^6^ Department of Land Resources Glenville State College Glenville WV 26351 USA; ^7^ Section of Plant Pathology and Plant–Microbe Biology School of Integrative Plant Science Cornell University Ithaca NY 14853 USA; ^8^ Department of Plant Biology University of Vermont Burlington VT 05405 USA

**Keywords:** belowground and aboveground interactions, context‐dependence, Ericaceae, ericoid mycorrhizae, floral traits, genotype‐specific effects, pollination, pollen limitation, symbiosis, *Vaccinium corymbosum*

## Abstract

**Premise:**

Most plants interact with mycorrhizal fungi and animal pollinators simultaneously. Yet, whether mycorrhizae affect traits important to pollination remains poorly understood and may depend on the match between host and fungal genotypes. Here, we examined how ericoid mycorrhizal fungi affected flowering phenology, floral traits, and reproductive success, among eight genotypes of highbush blueberry, *Vaccinium corymbosum* (Ericaceae). We asked three overarching questions: (1) Do genotypes differ in response to inoculation? (2) How does inoculation affect floral and flowering traits? (3) Are inoculated plants more attractive to pollinators and less pollen limited than non‐inoculated plants of the same genotype?

**Methods:**

To examine these questions, we experimentally inoculated plants with ericoid mycorrhizal fungi, grew the plants in the field, and measured flowering and floral traits over 2 years. In year 2, we conducted a hand‐pollination experiment to test whether plants differed in pollen limitation.

**Results:**

Inoculated plants had significantly higher levels of colonization for some genotypes, and there were significant floral trait changes in inoculated plants for some genotypes as well. On average, inoculated plants produced significantly larger floral displays, more fruits per inflorescence, and heavier fruits with lower sugar content, than non‐inoculated, control plants. Hand pollination enhanced the production of fruits, and fruit mass, for non‐inoculated plants but not for those that were inoculated.

**Conclusions:**

Our results demonstrate that inoculation with ericoid mycorrhizal fungi enhanced flowering and altered investment in reproduction in genotype‐specific ways. These findings underscore the importance of examining belowground symbionts and genotype‐specific responses in their hosts to fully understand the drivers of aboveground interactions.

Most plants engage with other plant competitors, herbivores, natural enemies, and pollinators aboveground, while engaging with a complex microbial community belowground. These above‐ and belowground interactions are most often examined separately and yet they may interact in complex and important ways that affect the traits and, ultimately, the fitness of the host plant. Moreover, the outcome of these interactions may depend on the genotypes of the interacting parties and affect plant traits and fitness in unpredictable and sometimes surprising ways.

As a premier example of these interactions, ca. 85% of terrestrial plants interact with fungi that live on or in roots and form mycorrhizae (Van der Heijden et al., 2015; Brundrett and Tedersoo, 2018). Mycorrhizae are among the oldest symbioses known and likely facilitated the colonization of terrestrial environments by plants ~450 million years ago (Ma) (Remy et al., 1994; Brundrett, 2002). In typical mycorrhizal interactions, partners exert direct, positive effects on one another, with plants translocating aboveground photosynthate in exchange for increased access to water and nutrients (Smith and Read, 2008). Although these reciprocal interactions typically accrue benefits to partners, the costs of association are variable, and there is growing evidence that the net outcome for both partners depends on the abiotic and biotic contexts in which they occur (Hoeksema et al., 2010; Johnson et al., 2010; Barber et al., 2013a), and on genotype‐by‐genotype compatibility (Rúa et al., 2016).

In addition to associating with mycorrhizal fungi, over 85% of all terrestrial flowering plants also require visits by animals to transfer pollen and effect fertilization (Ollerton et al., 2011). The importance of pollinators to the reproduction of most angiosperms is well known. Pollinators are essential to plant reproductive success and pollen limitation (or what is more often pollinator limitation) is common (Knight et al., 2005). There is some evidence that the symbiosis with mycorrhizal fungi can exert indirect effects on plant reproduction by enhancing floral traits important to pollinator attraction, including pollen production (Lau et al., 1995), nectar volume and/or sugar concentration (Gange and Smith, 2005), and inflorescence size (Wolfe et al., 2005). Mycorrhizal fungi may indirectly affect pollination success by altering floral traits that favor attractiveness to nonpollinating insects (Becklin et al., 2011). Mycorrhizae can also directly increase plant fitness by alleviating resource (water and nutrients) limitation, and if they affect traits that mediate interactions with pollinators (Gange and Smith, 2005; Barber and Soper Gorden, 2015), we might expect a complex suite of outcomes among plants, their fungal symbionts, and their pollinators.

The relative importance of resource limitation versus pollen limitation has received much attention in the literature because it gives us important insight about what is driving reproductive success and plant fitness (Knight et al., 2005; Maron et al., 2014). However, the degree to which plants are resource‐limited or pollinator‐limited may have as much to do with their interactions with mycorrhizal fungi as with the substrate in which they find themselves. For example, the allocation of photosynthetic carbon to mycorrhizal fungi may affect a plant's carbon allocation to sugars in nectar, while amelioration of drought by mycorrhizal fungi could affect nectar production. Last, if mycorrhizae affect plant investment in flower number or flower size, pollinator behavior will likely be altered as well. Several studies have examined the effects of mycorrhizal fungi on floral traits, pollinator behavior, and plant reproductive success (e.g., Cahill et al., 2008; Becklin et al., 2011; Barber et al., 2013b). In some cases, mycorrhizal fungi enhanced traits important to pollinator visitation (Gange and Smith, 2005); in others, mycorrhizal associations affected floral visitors in species‐specific ways (Becklin et al., 2011; Barber et al., 2013a) or altered community‐level responses of pollinators (Cahill et al., 2008; Bennett and Cahill, 2018). Thus, more work is required before a full understanding of the conditions under which mycorrhizae affect host interactions with pollinators and subsequent reproductive success will be reached (Barber et al., 2013a; Barber and Soper Gorden, 2015; Jin et al., 2015).

Here, we examined the benefit of mycorrhizae in a field setting where all was held constant except for whether plants were inoculated at planting and the genotype (cultivar) of the host, highbush blueberry (*Vaccinium corymbosum*; Ericaceae). Although cultivated blueberries are often provided fertilizer, the nutrient enhancement provided by mycorrhizae may be superior to that of fertilizer alone (Scagel, 2005), and plants may benefit in more subtle ways by association with mycorrhizal symbionts. Thus, understanding whether inoculation at planting alters traits important to pollinators and yield has basic as well as practical implications. We posed a series of questions that we addressed experimentally. Does inoculation of 2‐year‐old blueberry cuttings with ericoid mycorrhizal spores (1) enhance colonization over that of noninoculated plants 5 years after planting; (2) affect flowering phenology, floral morphology or floral display size; (3) alter patterns of pollen limitation; and (4) are responses specific to the host genotype? We examined the degree to which host genotype affects plant response to ErMF by comparing results among genetically distinct cultivars. We hypothesized that host genotypes would differ in responses to inoculation and that inoculation would alter floral traits important to visitation by pollinators. Overall, our results suggest that mycorrhizae directly enhance flowering, but their indirect effects on pollinators may be more subtle and due to changes in flower morphology that may affect floral visitors and their efficacy as pollinators.

## MATERIALS AND METHODS

### Study system

Ericaceous plants lack root hairs and are limited in their ability to extract N from the soil; they thus rely heavily on mycorrhizal partners for ca. 86% of N uptake (Johansson, 2000; Hobbie and Hobbie, 2006), P, and other scarce nutrients (Read, 1996; Kerley and Read, 1998; Vohník et al., 2012). *Vaccinium* species, in particular, harbor ericoid mycorrhizal fungi (hereafter “ErMF”), some of which have co‐evolved with their hosts for over 1 Myr (Cullings, 1996). Native *Vaccinium* rely on their mycorrhizal fungi (including ErMF) to capture scarce nutrients in often harsh soils (Read, 1996; Perotto et al., 2002).

Blueberries require insect pollinators to set fruit, and bees comprise more than 99% of all animals that visit blueberry flowers (Nicholson et al., 2017) providing substantial pollination services that improve both the quantity and quality of fruits (Stubbs and Drummond, 2001; Ehlenfeldt and Martin, 2007; Klein et al., 2007; Isaacs and Kirk, 2010). An array of native bees pollinates *Vaccinium* species, especially *Bombus* (Apidae), *Andrena* (Andrenidae), *Habropoda* (Apidae), *Lasioglossum* (Halictidae), and *Osmia* (Megachilidae) (Cane and Payne, 1988; Tuell et al., 2009; Hicks, 2011; Rogers et al., 2013; Nicholson et al., 2017). Honey bees (*Apis mellifera*) are often used as pollinators of commercial highbush blueberry, but most native bee visitors are superior pollinators on a per‐visit basis (Javorek et al., 2002; but see Benjamin and Winfree, 2014). In Vermont, where our study was done, native bumble bees (*Bombus* spp.) and large solitary bees (Andrenidae) are the most frequent visitors and primary pollinators of highbush blueberry, *V. corymbosum* (Nicholson et al., 2017; Nicholson and Ricketts, 2019).

### Experimental design

To examine whether inoculation with ErMF affects growth and flowering traits, and to assess whether inoculation with commercially available ericoid mychorrhizal fungi enhances fruit production and thus could be beneficial to farmers, we conducted a large‐scale, common garden experiment. In 2011, we planted 1100 2‐year‐old *V. corymbosum* bushes (Hartmann's Plant Co., Lakota, MI, USA) in a stratified random design in a 0.52‐ha hayfield at the Waterman Orchards in Johnson, Vermont (Adams soil series; https://soilseries.sc.egov.usda.gov/OSD_Docs/A/ADAMS.html). The certified organic farm includes 2 ha of blueberry plants, amongst a landscape of forests, hay fields, and low‐density residential development. At planting, 500 plants that included nine cultivars (Blue Crop, Blue Ray, Blue Jay, Bonus, Spartan, Duke, Nelson, Elliot, Toro) were treated with a peat moss‐based inoculum product containing *Hymenoscyphus ericae* (32 CFU/g) and *Oidiodendron griseum* (31,500 CFU/g) (BioTerra PLUS Ericoid Mycorrhizal Inoculant; Plant Health LLC, Corvallis, OR, USA), and 500 served as controls. An additional 100, noninoculated plants were planted as a buffer on the edges of the plot. The inoculum fungi are common symbionts of *Vaccinium* spp. (Couture et al., 1983; Dalpé, 2003) and were originally cultured from the roots of blueberry.

The treatment was applied by rubbing dry inoculum onto the wet root mass fully covering the roots, using ~0.10 kg of inoculum per plant (Waterman, 2014). Each of four blocks contained 10 rows of 25 plants per row with an additional row serving as a noninoculated buffer. We did not obtain the same number of plants per cultivar. Therefore, five of the nine cultivars were randomly assigned to occupy two rows of a single block each, while others were duplicated in more than one block. Plants were separated by 1.5 m, with inoculated and control plants of the same cultivar alternating in rows separated by 3 m. Root ball diameter, even in mature plants, rarely exceeds the diameter of the canopy. Thus, this spacing allowed ample growth of both roots and canopy without competition for nutrients or light. The area between rows was fully covered by grass and had a significantly higher pH (ca. 6.0 vs. 5.0), which provided a less hospitable border for shallow blueberry roots and ericoid mycorrhizae. Plants were treated annually with 80 kg/ha nitrogen from organic fertilizers, and weeds were controlled by addition of wood chip mulch and hand‐pulling.

### Mycorrhizal colonization

To confirm that the inoculation resulted in increased association with ErMF, we collected root samples from 3 to 10 plants per treatment (inoculated and controls) of each of six cultivars on 20 May 2015. We avoided sampling plants from one block because the soil was saturated with water in one corner and not representative of conditions in the other three blocks. To extract roots, we collected 2–3 soil cores ~5 cm from around the base of each plant using a 2.5 × 20 cm soil corer and combined samples of actively growing roots. In the laboratory, we washed soil from roots and cleared and stained samples using methods designed for mycorrhizal fungi (Brundrett and Abbott, 1994; Vierheilig et al., 1998) and modified slightly for blueberry as follows. Roots were placed in histology cassettes and autoclaved in 10% KOH for 45 min at 121°C, rinsed three times in distilled water, and then treated with H_2_O_2_ for 20 min at room temperature. Roots were again rinsed and then stained in a 5% ink and vinegar solution (v/v) while placed in a warm water bath for 24 h at 85°C. After staining, roots were rinsed for 20 min and stored in distilled water at 4°C until viewed. Fungal colonization of roots was scored using the magnified intersections method (McGonigle et al., 1990), examining ≥150 intersections for each sample. At each intersection, we classified root cell fungal status as: empty, occupied by ErMF by the presence of intracellular fungal coils, or containing dark septate endophytic fungi, an assemblage of fungi that also associate with blueberry roots.

### Inoculation, flowering, and floral traits

Flowering phenology and floral display size are often directly linked to pollinator attraction (Bauer et al., 2017; Munguía‐Rosas et al., 2017), and floral morphology can have important consequences for successful pollination of blueberries (Sampson et al., 2013). To determine whether inoculation affected flower production, phenology, and morphology, in 2015–2016 we sampled plants of eight cultivars. In 2015, flowering was limited by severe winter weather and deer herbivory; thus, we were only able to get floral measurements of five cultivars. Therefore, in analyses that included both years, we used data from only the five cultivars that were sampled in 2015 and again in 2016.

To assess flowering phenology and floral display size, we counted the number of flowers per inflorescence for at least 10 inflorescences per plant, estimated total inflorescences, and computed total flower production at the start of bloom and every 3–5 days thereafter. To assess whether inoculation affected floral morphology, we measured floral length from the base of flower corolla to its opening, maximum corolla width, and diameter of the floral opening, for 5 flowers per plant on 3–5 plants per treatment. Following Sampson et al. (2013), we estimated corolla size as the volume of a cylinder: mm^3^ = Corolla length × πr^2^, where *r* = corolla width/2.

### Inoculation and pollen limitation

To examine whether inoculated plants were less pollen‐limited than noninoculated controls, we conducted a pollen limitation experiment in 2016 using two cultivars (Duke and Blue Crop) for which plants were fully in bloom early in the season and were large enough to provide sufficiently well‐matched control and treatment inflorescences on each plant. For each of 10 plants per ErMF inoculation treatment, we chose four flowering branches as closely matched in phenology and number of inflorescences as possible. We randomly assigned two branches to be hand‐pollinated, and the other two branches served as open‐pollinated controls. For those assigned to the hand‐pollination treatment, we chose 3–5 inflorescences and, over the course of flowering, hand‐pollinated each open flower using pollen collected from multiple donors. Blueberries are “buzz‐pollinated”, requiring the vibration of a bees’ thoracic muscles to release pollen (Buchmann, 1983). To collect pollen, we used a “VegiBee” miniature electronic sonicator (VegiBee, Maryland Heights, MO, USA) that, when in contact with the flower, causes anthers to dehisce. We collected pollen into a microcentrifuge tube from flowers on multiple plants and then painted receptive stigmas with pollen using a small paintbrush. We handled flowers on control branches similarly but did not paint their stigmas with pollen. We later counted the number and weighed fresh mass of fruits produced, measured their sugar content with a refractometer (Dedej and Delaplane, 2004), and counted numbers of seeds and unfertilized ovules using the method of Desjardins and De Oliveira (2006). Due to difficulty in counting all flower buds during the flowering season, we could not accurately assess what proportion of flowers resulted in fruit production and instead analyzed total fruits per inflorescence as a measure of reproductive success.

### Statistical analyses

All analyses were conducted in R (R Core Team, 2018). We used analysis of variance to compare proportion of root cells that associated with ErMF between inoculated and control plants. We used linear mixed modeling to analyze effects of inoculation and pollen supplementation on floral and fruit traits using the package lme4 (Bates et al., 2016). We compared candidate models to select random and then fixed effects that minimized the Akaike information criterion (AIC) (Bolker et al., 2009). Candidate models were constrained to include inoculation treatment, cultivar, and their interaction (flower and inflorescence response variables), or these variables plus pollen supplementation treatment and their interaction (fruit yield variables in the pollen limitation experiment) as fixed effects. Other fixed effects considered in full models include year, block, and plant size (assessed as height × diameter). Cultivar, individual plant, and date were considered as random effects in the full models. After selecting a random effects structure, we compared 2–5 candidate fixed effects combinations, selecting the most parsimonious model that minimized AIC. Variables retained in best‐fit linear mixed effects models are described for each response variable below (see Results). We used the lmerTest package to calculate denominator degrees of freedom via the Satterthwaite approximation and *P*‐values for model parameters (Kuznetsova et al., 2017). To compare effects of ErMF treatment across multiple response variables for flowers and fruits, we computed coefficients of determination (semi‐partial and conditional *R* ^2^_glmm_) for fixed effects and full linear mixed models, respectively, using the package r2glmm, specifying the Nakagawa‐Schielzeth method (Nakagawa and Schielzeth, 2013).

## RESULTS

### Mycorrhizal colonization

**Figure 1:**
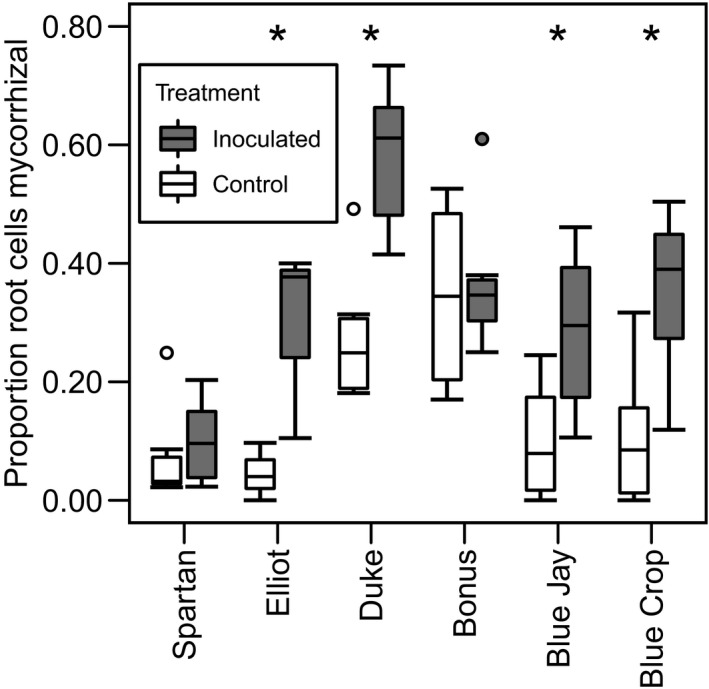
Inoculation with ericoid mycorrhizal fungi resulted in higher rates of root cortical cell colonization in highbush blueberry. This effect varied among cultivars and was statistically significant (asterisks) for four of six cultivars.

On average, 25.2% ± 0.02 SE (range: 0.0–73.4) of blueberry root cortical cells were colonized by ErMF. Root cells of treated plants were approximately twice as likely to be colonized by ErMF as those of controls, verifying that inoculation results in greater association with ErMF (*F*_1, 66_ = 36.48, *P* < 0.0001). Proportion of root cells inhabited by ErMF varied among cultivars (*F*_5, 66_ = 14.82, *P* < 0.0001), and there was a treatment × genotype interaction, revealing that inoculation resulted in increased ErMF colonization for four of six cultivars (*F*_5, 66_ = 3.27, *P* = 0.01; Fig. 1; Appendix S1).

### Inoculation, flowering, and floral traits

**Figure 2:**
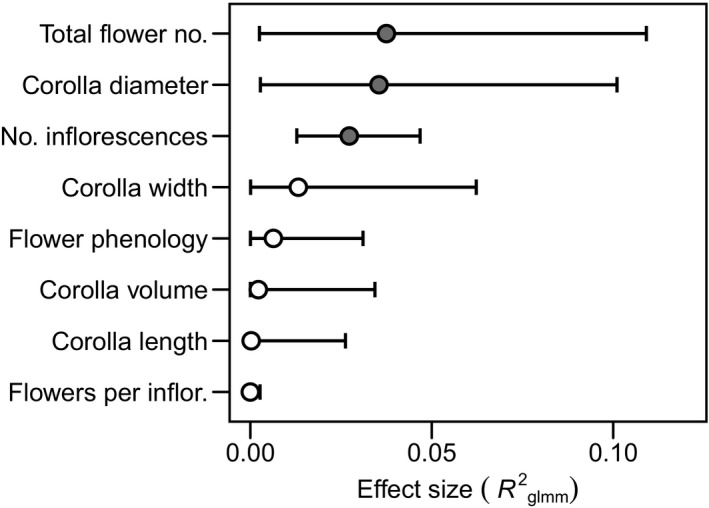
Effect sizes from linear mixed effects models analyzing effect of mycorrhizal inoculation treatment on blueberry reproductive traits. Effects are expressed as coefficients of determination (semi‐partial *R* ^2^_glmm_) ± 95% CI. Filled circles = statistically significant effects.

**Figure 3:**
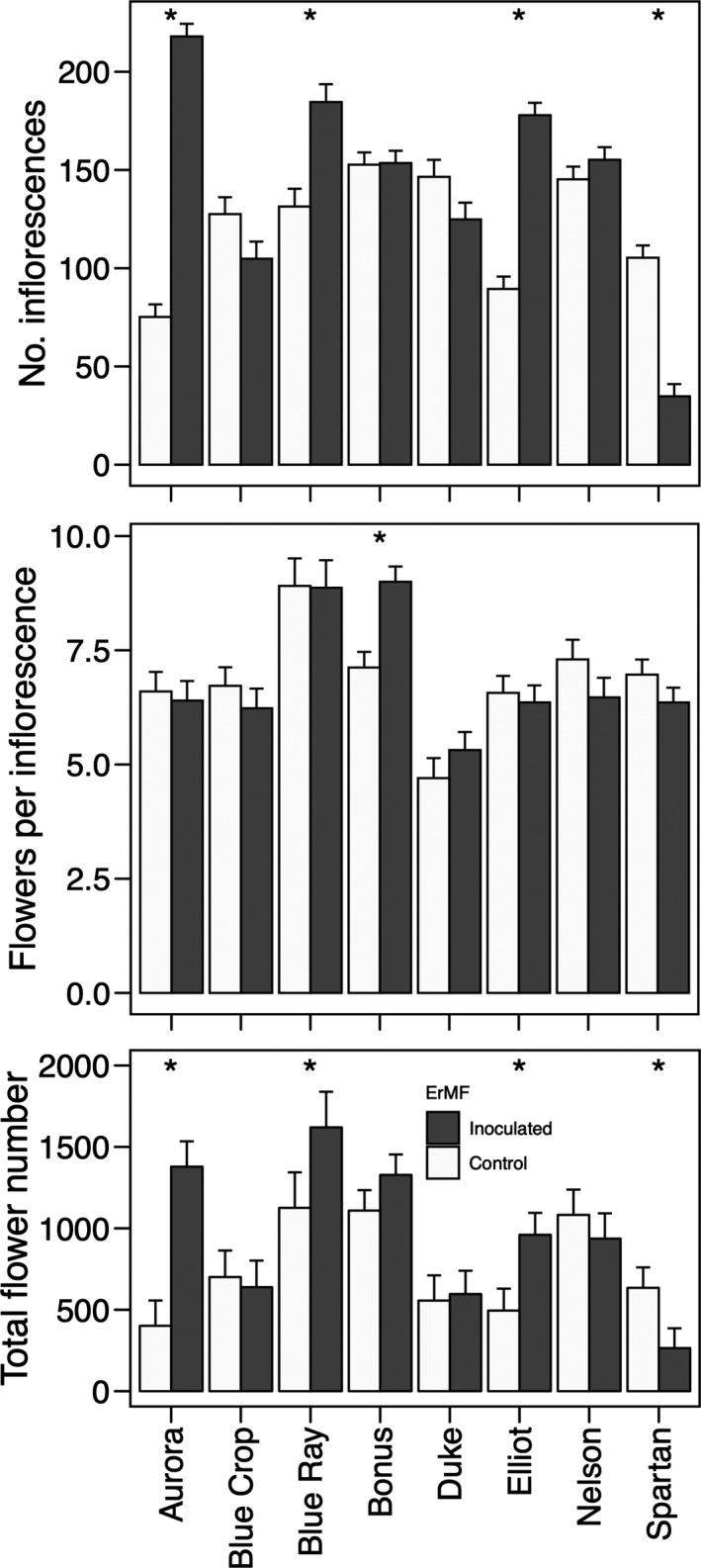
Least square means ± SE from linear mixed model analysis of floral display size, measured as numbers of inflorescences per plant, flowers per inflorescence, and total flowers per plant.

The best‐fit models explaining numbers of inflorescences, flowers per plant, and flowers per inflorescence included as fixed effects ErMF inoculation treatment, cultivar, and their interaction, as well as year of data collection. For flowers per inflorescence, the random effects retained in the best‐fit model were individual plant nested within cultivar, while models for numbers of inflorescences and flowers per plant included cultivar alone as a random effect. There was a significant positive effect of ErMF inoculation on the number of inflorescences produced by plants (*F*_1, 1280.9_ = 73.83, *P* < 0.0001; Fig. 2, Table 1) and overall floral display size (i.e., total flowers per plant; *F*_1, 90.7_ = 7.69, *P* = 0.007), but not on inflorescence size (i.e., number of flowers per inflorescence; *F*_1, 1820.5_ = 0.05, *P* = 0.83). Inflorescence number, size, and floral display size varied among genotypes (i.e., cultivars: *F* > 3.62, *P* < 0.0022; Fig. 3) and between years (*F* > 39.92, *P* < 0.0001), and for each, there was a significant treatment × genotype interaction, indicating differences among genotypes in their response to inoculation (*F* > 4.98, *P* < 0.0001).

**Table 1:** Flower and inflorescence traits measured over 2 years for eight blueberry cultivars after two ericoid mycorrhizal fungus inoculation treatments. Data are presented as least square means ± SE. Inoculation with mycorrhizal fungi had a statistically significant effect on inflorescence number, total flower production, and corolla opening diameter, but not on other response variables. There was a significant genotype × treatment interaction main effect for inflorescence number and size and flower number measures, with Tukey post hoc tests revealing significant effects (asterisks) of inoculation on some but not all cultivars. See Figure 3.

Cultivar	ErMF treatment	Diameter (mm)	Length (mm)	Width (mm)	Volume (mm^2^)	No. inflorescences	Inflorescence Size	No. flowers/plant
Aurora	Control	–	–	–	–	75.18 ± 6.41	6.60 ± 0.43	401.33 ± 155.44
	Inoculated	–	–	–	–	217.98 ± 6.41*	6.40 ± 0.43	1378.83 ± 155.44*
Blue Crop	Control	3.02 ± 0.14	10.56 ± 0.21	6.14 ± 0.19	315.18 ± 26.51	127.54 ± 8.58*	6.72 ± 0.41	701.12 ± 162.66
	Inoculated	2.98 ± 0.14	10.25 ± 0.21	5.74 ± 0.19	274.68 ± 26.51	104.83 ± 8.72	6.23 ± 0.43	639.04 ± 162.66
Blue Ray	Control	–	–	–	–	131.38 ± 9.06	8.91 ± 0.60	1125.63 ± 218.48
	Inoculated	–	–	–	–	184.58 ± 9.06*	8.87 ± 0.60	1620.03 ± 218.48
Bonus	Control	3.81 ± 0.12	11.50 ± 0.19	7.01 ± 0.17	445.96 ± 23.58	152.69 ± 6.25	7.12 ± 0.34	1108.96 ± 125.51
	Inoculated	3.75 ± 0.12	11.37 ± 0.19	6.89 ± 0.17	430.69 ± 23.71	153.55 ± 6.22	9.00 ± 0.33*	1328.16 ± 125.51
Duke	Control	5.35 ± 0.22	10.57 ± 0.33	8.76 ± 0.30	638.04 ± 41.97	146.50 ± 8.65	4.70 ± 0.44	556.24 ± 155.02
	Inoculated	5.20 ± 0.18	10.29 ± 0.27	8.91 ± 0.25	664.33 ± 34.70	124.83 ± 8.52	5.32 ± 0.39	596.53 ± 143.13
Elliot	Control	4.68 ± 0.18	11.51 ± 0.27	7.86 ± 0.24	559.27 ± 34.02	89.43 ± 6.31	6.57 ± 0.37	494.94 ± 134.69
	Inoculated	4.17 ± 0.18	12.10 ± 0.26	7.64 ± 0.24	559.05 ± 33.53	177.87 ± 6.31*	6.36 ± 0.37	960.48 ± 134.69*
Nelson	Control	–	–	–	–	145.25 ± 6.45	7.30 ± 0.43	1082.43 ± 155.44
	Inoculated	–	–	–	–	155.18 ± 6.41	6.47 ± 0.43	936.93 ± 155.44
Spartan	Control	4.49 ± 0.16	10.20 ± 0.24	8.29 ± 0.21	551.19 ± 30.21	105.34 ± 6.25*	6.97 ± 0.33	634.66 ± 125.51*
	Inoculated	4.11 ± 0.14	10.20 ± 0.21	8.11 ± 0.19	534.56 ± 26.20	34.79 ± 6.22	6.36 ± 0.32	264.55 ± 121.79

Final models that minimized AIC for flower dimension measures each included ErMF inoculation treatment, cultivar, and their interactions fixed effects and plant nested within cultivar as a random effect. Inoculated plants produced flowers with opening diameter of the corolla 2.8% smaller than that of control plants (*F*_1, 40.6_ = 5.05, *P* = 0.03), but inoculation had no effect on corolla length, width, or volume (*F* < 1.27, *P* > 0.27; Fig. 2, Table 1). Although each of these flower dimensions varied among genotypes (*F* > 18.77, *P* < 0.0001), we found no significant treatment × genotype interactions (*F* < 1.01, *P* > 0.41). Corolla dimensions were positively correlated with each other (data not shown).

### Inoculation and pollen limitation

**Figure 4:**
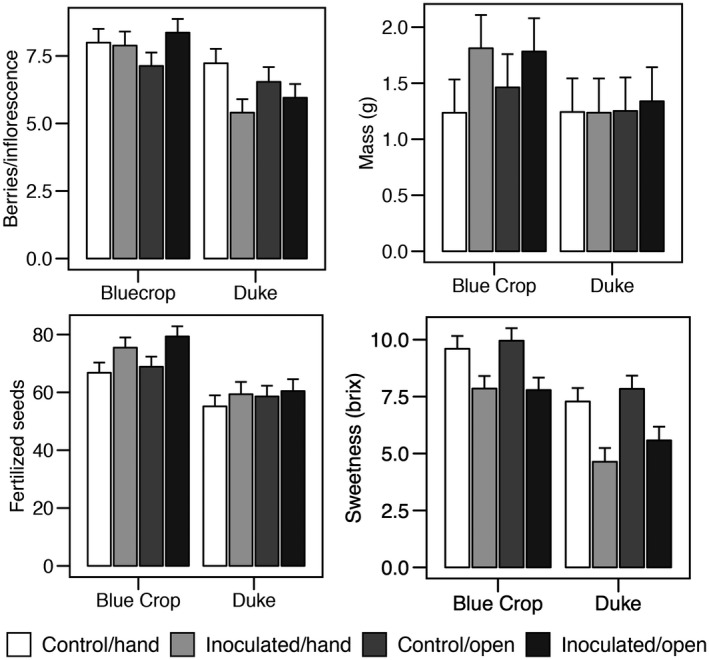
Least square means ± SE from linear mixed model analysis of blueberry reproduction as a function of cultivar (genotype), and mycorrhizal inoculation (“ErMF”) and pollen supplementation (“pollen addition”) treatments. Inoculation significantly increased fruit mass (*F*_1, 35.6_ = 6.55, *P* = 0.01) and reduced sugar concentration (*F*_1, 34.4_ = 16.64, *P* = 0.0003). Pollen addition significantly reduced the number of fertilized seeds (*F*_1, 444.5_ = 7.07, *P* = 0.008), and for berries produced per inflorescence, there was a significant ErMF × pollen addition effect (*F*_1, 122.62_ = 3.93, *P* = 0.05). See Table 2 for post hoc comparisons demonstrating genotype‐level differences in responses to ErMF inoculation and pollen deficit.

Best‐fit models for the response variables in the pollen‐limitation experiment were constrained to include as fixed effects ErMF and pollen addition treatments, cultivar, and all two‐ and three‐way interactions among them. As random effects, these models included individual plant nested within cultivar. The number of fruits per inflorescence was not affected by ErMF (*F*_1, 40.4_ = 0.54, *P* = 0.47) or pollen addition treatments (*F*_1, 122.6_ = 0.17, *P* = 0.68; Fig. 4, Table 2). Hand‐pollinated inflorescences of noninoculated plants produced more fruits than did the other treatment groups, driving a significant inoculation × pollen addition interaction (*F*_1, 122.62_ = 3.93, *P* = 0.05). Individual fruit mass was larger on plants inoculated with ErMF (*F*_1, 35.6_ = 6.55, *P* = 0.01), but there was no effect of pollen supplementation (*F*_1, 445.5_ = 3.57, *P* = 0.06) and no significant interaction between the two treatments (*F*_1, 445.6_ = 2.75, *P* = 0.10). The number of fertilized seeds was lower in fruits from hand‐pollinated flowers (*F*_1, 444.5_ = 7.07, *P* = 0.008), but there was no effect of inoculation on seed number (*F*_1, 38.2_ = 3.22, *P* = 0.08) and no significant interaction between inoculation and hand pollination (*F*_1, 444.5_ = 0.03, *P* = 0.87) on seed number. Fruits of inoculated plants had lower percentage sugar than those of control plants (*F*_1, 34.4_ = 16.64, *P* = 0.0003), but there was no effect of pollen addition (*F*_1, 478.3_ = 2.71, *P* = 0.10) or an interaction among treatments on sugar content (*F*_1, 478.1_ = 0.10, *P* = 0.75; Fig. 4, Table 2).

**Table 2:** Least square mean ± SE number of fruits per inflorescence, fruit mass, seed number, and fruit sweetness in two blueberry cultivars in an experiment crossing ErMF inoculation treatment with a pollen supplementation treatment. Asterisks indicate means that are statistically significantly higher than their pair (*P < 0.05; **P < 0.01; ***P < 0.001). See Figure 4.

Effect	Cultivar	Pollen	ErMF	Mean ± SE
Fruit number	Blue Crop	Hand	Control	7.99 ± 0.51
			Inoculated	7.88 ± 0.52
		Open	Control	7.13 ± 0.5
			Inoculated	8.36 ± 0.51
	Duke	Hand	Control	7.23 ± 0.53*
			Inoculated	5.4 ± 0.5
		Open	Control	6.54 ± 0.55
			Inoculated	5.95 ± 0.51
Fruit mass (g)	Blue Crop	Hand	Control	1.24 ± 0.3
			Inoculated	1.81 ± 0.3***
		Open	Control	1.46 ± 0.3
			Inoculated	1.78 ± 0.3*
	Duke	Hand	Control	1.24 ± 0.3
			Inoculated	1.24 ± 0.31
		Open	Control	1.25 ± 0.3
			Inoculated	1.34 ± 0.3
No. fertilized seeds	Blue Crop	Hand	Control	66.77 ± 3.53
			Inoculated	75.45 ± 3.52
		Open	Control	68.86 ± 3.5
			Inoculated	79.34 ± 3.5*
	Duke	Hand	Control	55.16 ± 3.79
			Inoculated	59.36 ± 4.24
		Open	Control	58.59 ± 3.71
			Inoculated	60.45 ± 4.1
Fruit sweetness (brix)	Blue Crop	Hand	Control	9.6 ± 0.56*
			Inoculated	7.85 ± 0.55
		Open	Control	9.95 ± 0.55**
			Inoculated	7.79 ± 0.55
	Duke	Hand	Control	7.28 ± 0.59**
			Inoculated	4.64 ± 0.6
		Open	Control	7.84 ± 0.58**
			Inoculated	5.58 ± 0.6

## DISCUSSION

Most plants associate with mycorrhizal fungi and animal pollinators, which can have myriad and profound effects on their hosts. Here, inoculation affected flowering traits potentially important to pollinator attraction and reproductive success in genotype‐specific ways. For some genotypes, inoculated plants had more inflorescences per plant and more flowers, thus producing significantly larger overall floral displays than their noninoculated counterparts.

Given that inoculation enhanced floral display, we predicted that inoculated plants would attract more pollinators and thus be less pollen‐limited than their noninoculated neighbors. Floral display size is often positively correlated with pollinator visitation rates and resulting plant reproductive success (Mitchell et al., 2004; Karron and Mitchell, 2012; Bauer et al., 2017; Munguía‐Rosas et al., 2017). Pollen limitation was revealed in other studies of highbush blueberry in the area around which we worked (Nicholson and Ricketts, 2019), and plants with larger floral displays would be expected to be more attractive to pollinators and thus less pollen‐limited. Although both inoculation and pollen supplementation increased the number of fruits produced, each did so only in the absence of the other. And, contrary to our expectation, pollen supplementation reduced rather than increased one measure of reproductive success—the production of fertilized seeds. Previous studies have also found reduced seed set with hand pollination and suggested several causes (Ashman et al., 2004). These include unintentional “error” such as the clogging of stigmatic surfaces with incompatible pollen and/or inadvertent damage to stigmas, and more biologically interesting ones like plants’ ability to make fewer but better seeds, or the balance between resource and pollen limitation (see Ashman et al., 2004 for review).

Although highbush blueberry is self‐compatible, it often benefits from receiving outcross pollen (Dogterom et al., 2000). However, it is possible that not all genotypes (cultivars) are equally compatible and that we inadvertently introduced a large fraction of incompatible pollen by hand. Indeed, hand outcrossing of one of the varieties we used, Blue Crop, can reduce berry production (Ehlenfeldt, 2001), although this effect appears to be variable and was not seen by others (Dogterom et al., 2000). We collected pollen from dozens of flowers of several plants of different cultivars into a single collection tube or petri dish. Bees, on the other hand, most often moved from flower to flower within plants and between adjacent plants before flying away. Thus, bees may have been more likely to transfer pollen within the same cultivar than we were, and they may deliver less pollen per visit. One or both differences in natural versus hand pollination could have contributed to our results.

Inoculation reduced one measure of flower size, corolla opening diameter, but did not affect other aspects of floral morphology. Although the effect on corolla diameter was modest, it could affect the suite of insects that can access floral resources (Courcelles et al., 2013; Sampson et al., 2013) and thus plant reproductive success. Honey bees, which do not sonicate the flowers while foraging and are relatively poor pollinators of blueberry (Javorek et al., 2002; but see Benjamin and Winfree, 2014), might be restricted from entering flowers with more narrow openings. Short‐tongued native bees might be similarly restricted from accessing nectar in this way. Bumble bees, on the other hand, can gather and transfer pollen without entering the flower and are highly efficient pollinators of blueberries (Nicholson and Ricketts, 2019). Thus, when bumble bees are numerous enough to ensure most flowers are pollinated, the restriction of honey bees and short‐tongued bees may benefit the plant by reducing the waste of floral rewards. Others, too, have found that the interactions among mycorrhizal fungi and their hosts alter interactions with floral visitors in species‐specific ways, with some floral visitors responding strongly to changes driven by the symbioses and others responding little or not at all (Gange and Smith, 2005; Cahill et al., 2008; Becklin et al., 2011; Barber et al., 2013b). These, along with our results, suggest that the outcome of plant response to mycorrhizal fungi will depend on the context of other species with which the host plants interact.

We predicted that inoculated plants would have greater reproductive success than controls due to enhanced resource acquisition by mycorrhizae, an effect that would be particularly strong in flowers benefiting from pollen supplementation. Inoculated plants produced larger fruits but did not produce more fruits per inflorescence than the noninoculated controls. Hand pollination, however, had no effect on either fruit set or fruit size except in the absence of inoculation. Perplexingly, hand‐pollinated inflorescences of noninoculated plants produced more fruits than open‐pollinated inflorescences of inoculated plants. These results suggest that mycorrhizae may have exerted a direct effect of enhancing plant access to resources but with a corresponding trade‐off between early season investment in flowers and later season investment in fruits. *Vaccinium* pre‐forms buds in the fall. Thus, inoculation appears to have allowed plants to invest more in flower buds but not to fully sustain that investment through fruiting. The cost of association with mycorrhizal fungi has been identified by others as well and points to the complex nature of these interactions (Johnson et al., 1997; Becklin et al., 2011; Kiers et al., 2011).

In contrast to our results, hand pollination of Blue Crop blueberry bushes significantly increased seed set, fruit set, and fruit size on other farms in other years in the same area (Nicholson and Ricketts, 2019). Nicholson and Ricketts (2019) pointed out, however, that the overall trends obscure considerable among‐farm variation in the levels of pollen limitation—with plants at some farms experiencing a high degree of pollen limitation, while others experienced lower levels or none at all. The degree to which plants are pollen‐limited will depend on the abundance and identity of pollinators in each year and site (Nicholson et al., 2017). Although we did not quantify visitation rates in 2015, bees were abundant. Yet, hand‐pollinated plants produced more and bigger fruits but only in the absence of inoculation. That we did not see significant pollen limitation for the inoculated plants may be have been driven more by alterations in resource acquisition and allocation than by pollen limitation.

Our results suggest complex ways in which mycorrhizal fungi affect plant traits and fitness. Enhancement of resources is the most obvious direct effect of mycorrhizae—although that benefit may be realized only when nutrients are limiting and thus highly dependent on soil conditions, competition with other plants, and the life‐ or reproductive‐stage of plant hosts. Indirect effects, such as alterations of flower size that ease access by some floral visitors while reducing access by others, may benefit plants when floral visitors are abundant and the ability to allow access by the most effective pollinators is better than allowing access by all. However, we were unable to observe pollinators long enough to determine whether they behaved differently toward inoculated versus noninoculated plants; thus, we cannot conclude whether the differences we found in floral morphology affected visitation rate or floral access.

We assumed some degree of pollen limitation based on evidence from other farms nearby (Nicholson and Ricketts, 2019). Our assumption was upheld in finding enhanced fruit production on branches that were hand‐pollinated. Yet, that is an indirect measure of pollinator visitation and behavior. In a subsequent study using potted plants, we found that bees spent a significantly longer time at flowers of inoculated plants than those of controls (L. L. Richardson, personal observation). We observed this effect for both efficient and inefficient pollinators (mining bees of the genus *Andrena* and honey bees, respectively), and speculate the mechanism could be related to altered floral attractiveness operating at a bee species‐specific level, as observed in other mycorrhizae studies (Barber et al., 2013a, 2013b). In our study using potted plants, inoculation and its concomitant changes in floral traits reduced pollen limitation. Thus, it is likely that the greater time spent at flowers of inoculated plants resulted in higher reproductive success. Further research is needed to clarify how mycorrhizae affect floral visitors most important to plant reproduction and thus exerting the strongest selection on floral traits and, perhaps, on association with mycorrhizal fungi.

Here, we found differences in responses to inoculation among host genotypes—a finding consistent with those of others in suggesting that the match between fungal symbionts and their hosts affects the outcome of the interaction (Klironomos, 2003; Scagel, 2005; Cahill et al., 2008; Rúa et al., 2013, 2016; Middleton et al., 2015; Bennett and Cahill, 2018). Inoculation significantly increased root colonization in four of the six genotypes for which we had multiple years of data. Cultivar (i.e., genotypes) specific effects of inoculation were found by others as well (Scagel, 2005; Scagel and Yang, 2005). Early‐blooming cultivars tended to have higher levels of colonization than later‐blooming ones (Scagel and Yang, 2005) and colonization altered the ways in which plants utilize nutrients (Powell and Bates, 1981; Yang et al., 2002; Scagel, 2005; Zinati et al., 2011). Even closely related genotypes showed a high degree of variability in response to inoculation. For some, inoculation enhanced the production of inflorescences and flowers, while in others plants accumulated nutrients without increasing growth or reproduction (referred to as luxury consumption; Scagel, 2005). In our study, one cultivar, Spartan, showed a decrease in both flower and inflorescence number, suggesting that the ErMF we used does not form a beneficial interaction with all blueberry genotypes, at least in this field setting. Other genotypes, such as Duke, appeared to have no response to the ErMF inoculum we used for inflorescence or flower production (Fig. 3), fruit number, berry mass, or number of fertilized seeds (Table 2), in contrast to the strong positive response of Blue Crop for the berry traits. A lack of responsiveness to mycorrhizal partners is often seen in domesticated crops in contrast to their wild ancestors and may suggest a decreased reliance on mycorrhizal associations (Martin‐Robles et al., 2018). The cultivar‐specific differences in responsiveness to inoculation, and their underlying causes, remain an area ripe for investigation.

Such differences are not unique to blueberry. In maize (*Zea mays*) and chickpea (*Cicer arietinum*), different genotypes showed a range of responses to mycorrhizal partners (Sawers et al., 2017; Bazghaleh et al., 2018). For maize, these differences were expressed in a more than 2‐fold increase in shoot dry mass in some genotypes over others (Sawers et al., 2017), while in chickpea all genotypes responded positively to inoculation with arbuscular mycorrhizal fungi (AMF) but not to coinoculation with AMF and endophytes (Bazghaleh et al., 2018). A comparison of the mycorrhizal responsiveness of 27 modern crops versus their wild ancestors revealed that the wild ancestors benefited from mycorrhizal associations irrespective of soil phosphate conditions, whereas the more modern genotypes did not (Martin‐Robles et al., 2018). Together, these studies and ours underscore that intraspecific variation in the host genotype affects interactions with mycorrhizal partners.

Although we found significant differences in colonization between inoculated and noninoculated plants, the lack of strong differences in some of the response variables we measured could be due, in part, to colonization of noninoculated plants. All plants were planted as 2‐year‐old seedlings that were originally grown in peat moss that likely contains some mycorrhizal fungi. Therefore, it is likely that all plants hosted fungal symbionts before planting. In addition, there are other Ericaceae in the area, and thus, it is likely that the field harbored ericoid mycorrhizal fungi. Regardless, if our inoculation treatment had been swamped out by either of these, we would expect to see no differences between treatments. Yet, we did. There may, however, have been priority effects such that fungi already living in the roots of the plants at purchase could have excluded those of the inoculum. If so, that would indicate a stronger relationship between some host plant genotypes and their mycorrhizal symbionts than in others and would underscore the genotype‐specific nature of the interaction (Rúa et al., 2016 and references therein). Ideally, we would have measured colonization on each of the same plants for which we measured flowering and floral traits. Our lack of doing so compromised our ability to tightly link colonization of an individual plant with the traits we measured. Future work that links the identity of the fungi with host genotype and phenotype, and molecularly characterizes the fungi inhabiting different genotypes, will greatly advance our knowledge of fungal functional diversity and its effects (Leopold, 2016).

Our field results found weaker effects of ErMF than in other work (Koron and Gogala, 1998; Scagel, 2005) using potted plants, which could be due to a variety of mechanisms. When highbush blueberry plants were inoculated with ericoid fungal spores, they responded positively and grew faster (Koron and Gogala, 1998; Scagel, 2005). In an experiment similar to ours, using the same inoculum preparation, plants grown in pots produced larger flowers and berries (L. L. Richardson, T. H. Ricketts and A. K. Brody, unpublished data). We might attribute the differences seen in pot experiments versus our field experiment to a variety of mechanisms. For one, in more natural field conditions, the positive effects of inoculum may be overshadowed by competition and colonization by other fungi and nonfungal microbes. We know that many hundreds of other fungi are found on and in the roots of these plants. Using high‐throughput sequencing of the ITS region, we discovered differences in fungal assemblages between our inoculated and noninoculated treatments along with the species present in the inoculum (A. K. Brody, J. M. Harris and L. L. Richardson, unpublished data). In addition, the soil and nutrient status of plants grown in pots versus those in the field is likely to differ dramatically, which will, in turn, affect plant response to mycorrhizal fungi (Scagel, 2005; Barber et al., 2013b; Van der Heijden et al., 2015). One motivation for our study was to understand whether, under field‐realistic conditions, inoculation would enhance fruit production and thus could be used to benefit farmers. Our finding that, on average, inoculation increased the number of fruits per inflorescence and berry size suggests that ErMF may be beneficial but in genotype‐specific ways and only under conditions in which the fungi do not compete with the host for resources. Thus, the benefit of inoculating plants will depend on the host cultivar and farming practices (also see Scagel, 2005). In future work, we will explore the links between fungal assemblages and functional trait differences, as well how soil conditions alter the functional link between ericoid mycorrhizae, other fungal symbionts, and their host plants.

The indirect effects of mutualistic partners can often drive the outcome of selection (Guimarães et al., 2017). Thus, illuminating the ways in which below‐ and aboveground partners interact can reveal targets of selection and provide insight into how variation is maintained in strong, reciprocal, two‐species interactions (Wardle et al., 2004; Barber and Soper Gorden, 2015). Our results show that the association of plants with their mycorrhizal symbionts can be important to traits formerly assumed to be driven strictly by interactions with pollinators. Moreover, the effects of these associations on floral traits were dependent on the genotype of the host plant and may be dependent on the genotype of the fungal symbionts as well. Our results add to the growing but still incomplete knowledge of how belowground interactions affect those aboveground and offer exciting avenues for further research.

## AUTHOR CONTRIBUTIONS

A.K.B., B.W., T.H.R., A.L.D., J.M.H. and L.L.R. planned and designed the research. A.K.B., B.W., J.B.G. A.L.D., J.M.H. and L.L.R. conducted fieldwork and laboratory work. L.L.R. analyzed the data. A.K.B. and L.L.R. wrote the first draft of the manuscript and B.W., T.H.R., A.L.D., J.B.G., and J.M.H. contributed substantive revisions.

## Supporting information


**APPENDIX S1.** Mycorrhizal colonization among cultivars.

## Data Availability

Data available from the Dryad Digital Repository: https://doi.org/10.5061/dryad.nf5nk30 (Brody et al., 2019).
