# Supplementary material for: Genotype‐specific effects of ericoid mycorrhizae on floral traits and reproduction in Vaccinium corymbosum
Source: Am J Bot. 2019 Nov 1;106(11):1412–22. doi: 10.1002/ajb2.1372 (PMC6899715; doi:10.1002/ajb2.1372)
Supplement: Supplementary file 1 — APPENDIX S1. Mycorrhizal colonization among cultivars. [file AJB2-106-1412-s001.docx]

**Appendix S1.** Mycorrhizal colonization among cultivars.

Inoculation with mycorrhizal fungi resulted in increased association between blueberry plant root cortical cells and ErMF. Cultivars varied in the strength of this association, and there was a significant cultivar × genotype interaction main effect, with Tukey post hoc tests revealing significant effects (asterisks) of inoculation on four of six cultivars. See main text Figure 1.

| Cultivar | ErMF treatment | *n* | Proportion colonized |
| --- | --- | --- | --- |
|  |  |  | Mean ± SE |
| Blue Crop | Control | 6 | 0.11 ± 0.05 |
|  | Inoculated | 6 | 0.35 ± 0.06* |
| Blue Jay | Control | 6 | 0.10 ± 0.04 |
|  | Inoculated | 6 | 0.29 ± 0.06* |
| Bonus | Control | 6 | 0.35 ± 0.07 |
|  | Inoculated | 6 | 0.37 ± 0.05 |
| Duke | Control | 6 | 0.28 ± 0.05 |
|  | Inoculated | 6 | 0.58 ± 0.05* |
| Elliot | Control | 3 | 0.05 ± 0.03 |
|  | Inoculated | 3 | 0.29 ± 0.10* |
| Spartan | Control | 6 | 0.08 ± 0.04 |
|  | Inoculated | 6 | 0.10 ± 0.03 |
